# Evaluating a Smoothie-Based Nutrition Education Program to Improve Nutrition Security in Rural Adolescents

**DOI:** 10.3390/nu18020305

**Published:** 2026-01-19

**Authors:** Amelia Sullivan, Emma Watras, Bryn Kubinsky, Kathyrn Yerxa, Kayla Gayer, Elizabeth Hufnagel, Kathleen A. Savoie, Jade McNamara

**Affiliations:** School of Food and Agriculture, University of Maine, Orono, ME 04469, USA; amelia.sullivan@maine.edu (A.S.); emma.watras@maine.edu (E.W.); bryn.kubisnky@maine.edu (B.K.); kate.yerxa@maine.edu (K.Y.); kayla.gayer@maine.edu (K.G.); elizabeth.hufnagel@maine.edu (E.H.); ksavoie@maine.edu (K.A.S.)

**Keywords:** nutrition security, rural health, adolescence, choice architecture, social cognitive theory, fruit consumption, school nutrition, skin carotenoids, diet quality, National School Lunch Program

## Abstract

**Background/Objective:** Nutrition security, defined as consistent access to and consumption of nutritious foods that support health, remains a persistent challenge in rural populations. The HEALTHY (Helping Early Adolescents Live Their Healthiest Youth) program aimed to improve rural adolescents’ nutrition security through school-based strategies. This study evaluated its effectiveness by examining changes in fruit consumption, fruit waste, and skin carotenoid levels. **Methods:** A quasi-experimental, pre–post program was assessed in five rural middle schools (two experimental sites, three comparison sites). The programming paired four biweekly smoothie taste tests with nutrition education grounded in Social Cognitive Theory and Choice Architecture. Students in grades 3–8 (N = 149) participated. Digital tray photographs quantified selection and waste. The Veggie Meter^®^ assessed skin carotenoids on a scale from 0 to 800. Surveys captured perceptions and self-reported intakes. Analyses included *χ*^2^, McNemar’s, GLMM, paired *t*-tests, and ANCOVA. Significance was set at *p* < 0.005. **Results:** At post-program, 98.3% of experimental trays contained the standard fruit option and/or a smoothie, compared with 41.0% of comparison trays (*χ*^2^ = 41.66, *p* < 0.001). Fruit selection odds were 16.22 times higher in experimental schools (95% CI: 6.30–41.77, *p* < 0.001). Among trays with both (n = 39), smoothie waste was lower than the standard fruit option waste (*t*(38) = −7.10, *p* < 0.001, *d* = 1.14), resulting in greater estimated consumption (~0.43 vs. ~0.15 cups). Skin carotenoids increased in both groups, with greater improvement among experimental students in the lowest baseline quartile, *F* (1,19) = 9.20, *p* = 0.007, partial *η*^2^ = 0.326. **Conclusions:** The HEALTHY program, which paired frozen-fruit smoothies with nutrition education, may offer a feasible and scalable approach to improving nutrition security among rural adolescents.

## 1. Introduction

Adolescence is a pivotal period for shaping lifelong health trajectories [1,2]. However, diet quality during this stage often falls short of recommended guidelines [3,4,5,6]. Fruits provide essential vitamins, minerals, fiber, and phytochemicals that support healthy development and reduce chronic disease risk [7,8,9]; yet national surveys indicate that only about one-third of adolescents, aged 12–19, consume whole fruit on a given day in the United States (U.S.) [10,11]. Improving adolescents’ diet quality is therefore central to advancing nutrition security in the U.S., which encompasses not only food access but also consistent availability, affordability, and consumption of nutritious foods that support growth and long-term health [12].

The National School Lunch Program (NSLP), which served approximately 29 million U.S. children daily as of 2024, represents a critical setting for addressing nutrition security challenges [13,14]. Despite the NSLP’s requirement for daily fruit offerings, provision alone does not guarantee consumption, with 20 to 40% of school-served fruit being wasted [15,16,17,18,19]. High levels of fruit waste reduce the nutritional impact of federally reimbursed meals, strain school budgets, and contribute to inefficient use of food resources [20]. These concerns are magnified in rural schools where budgets are often constrained, and food service directors must balance rising costs with limited staff and infrastructure [21].

Rural communities face geographic and seasonal barriers that exacerbate disparities in fruit access, consumption, and overall nutrition security. Transportation constraints, socioeconomic factors, and limited seasonal availability restrict access to affordable produce. For rural schools, these barriers make it especially difficult to ensure a consistent year-round supply of high-quality fruit options [21,22,23,24,25,26,27]. Rural disparities illustrate that nutrition security is not solely a matter of individual choice but is also shaped by structural and environmental barriers. Programming that strengthens both the availability and acceptability of healthy foods within existing systems, such as the NSLP, is therefore central to improving nutrition security in rural populations.

Preliminary work with rural adolescents identified frozen-fruit smoothies as a desirable and feasible strategy for increasing fruit intake. Students expressed enthusiasm for the taste, variety, and freshness of the smoothies [28,29]. Frozen fruit is nutritionally comparable to fresh fruit and is available year-round with less spoilage [30,31,32]. As a result, frozen fruit offers a promising strategy to increase access to and consumption of fruit, reduce waste, and support nutrition security in rural school systems.

Strategies to improve dietary behaviors are most impactful when guided by education and behavioral theory [33,34,35,36]. Social Cognitive Theory (SCT) emphasizes personal agency and learning through experience. Key constructs include self-efficacy, behavioral capability, and observational learning, all of which align with adolescents’ interest in experiential learning and hands-on food experiences [37,38,39]. Choice Architecture, which draws from Behavioral Economics, focuses on how subtle environmental cues influence decision-making. Techniques such as increasing visibility, improving convenience, and optimizing placement of healthy options can shape food choices in predictable ways [40,41,42,43,44]. When used together, SCT and Choice Architecture support both individual behavior changes and environmental shifts within school meal programs, making them appropriate foundations for testing frozen-fruit smoothies as a dual strategy that serves as both a nutritious menu item and a behavior change tool [45].

Although previous school-based programs have used behavior theory-driven or fruit-focused approaches independently [46,47,48,49], none have combined these elements. Moreover, no study has integrated a frozen-fruit smoothie-based educational program designed with both SCT and Choice Architecture in rural middle schools. In addition, while interest in using pressure-mediated reflection spectroscopy to analyze skin carotenoid levels in adolescent nutrition research has grown [50], most studies have been cross-sectional or single-group designs [51]. To our knowledge, only two studies have used a comparison group design to validate changes in fruit and vegetable intake among adolescents using this biomarker [52,53]. Skin carotenoid levels offer a non-invasive, reliable indicator of carotenoid-rich food intake (e.g., fruit) and fill an important gap in the literature by providing an objective measure of dietary intake [54,55].

Considering the high levels of fruit waste and the disparities rural schools face, research that integrates behavioral theory, system-level considerations, frozen fruit availability, and objective dietary assessment is needed to inform scalable program approaches. To address this gap and to examine the potential benefits of integrating frozen-fruit smoothies into the NSLP, the present study assessed the HEALTHY (Helping Early Adolescents Live Their Healthiest Youth) program, which included four smoothie-based educational sessions delivered every other week over eight weeks to promote fruit consumption and reduce fruit waste among rural adolescents. We hypothesized that, relative to comparison schools, experimental schools would exhibit significantly higher fruit consumption, lower fruit waste, and improved skin carotenoid levels following the programming.

## 2. Materials and Methods

### 2.1. Study Design

This quasi-experimental study employed a pre–post design to evaluate the HEALTHY program among rural adolescents. Implemented over eight weeks, the programming included four smoothie-based educational sessions delivered every other week. The primary summative outcomes outlined in this study include digital tray photography to quantify waste and consumption, skin carotenoid levels assessed with reflection spectroscopy, and paper surveys collecting demographics, self-reported perceived dietary intake frequency, and perceptions of the school food environment.

The programming was implemented from February to April 2025, with pre-program data collection in January 2025 and post-program data collection in May 2025. Two schools participated as experimental sites, and three served as comparison sites. The University of Maine’s Institutional Review Board (IRB) approved this study and deemed it exempt from full board review.

### 2.2. Participants and Recruitment

Schools affiliated with the Maine Farm and Sea to School Institute (ME-FSTSI) were recruited, leveraging established connections from the research team’s prior collaborations with these sites [56]. In fall 2024, researchers sent informational invitations to nine ME-FSTSI schools via email. Researchers then invited interested schools and their administrators (e.g., principals, superintendents, and nutrition directors) to participate in 45 min virtual meetings with the research team to discuss study procedures, logistics, and expectations. We aimed to recruit six schools, three serving as experimental and three serving as comparison. Five rural schools enrolled, with two designated as experimental sites and three serving as comparison sites.

Upon confirmation that schools would participate, the research team purposively assigned schools into the experimental and comparison designations. Schools were not randomly assigned; rather, schools that expressed interest and provided administrative willingness and approval to host the HEALTHY programming within existing cafeteria operations were designated as experimental sites, while participating schools that did not host the programming served as comparison sites. District-level data from the National Center for Education Statistics better contextualizes participating schools. The two experimental schools were located in small, rural, remote districts, whereas the comparison schools were drawn from somewhat larger districts spanning suburban communities. All schools were located in the most rural state in the U.S.

Students in grades three through eight were eligible, as these grades shared lunch periods in schools where the cafeteria-based program was delivered. Schools distributed the parental consent letter through newsletters and electronic communication platforms and shared a short video introducing the research team and outlining the study objectives and procedures. Parents provided electronic consent via Qualtrics, and on data collection days, trained research assistants (RAs) obtained verbal assent from students before participation. A total of 149 students enrolled across the five participating schools.

### 2.3. Program Design

The HEALTHY program consisted of four frozen-fruit smoothie-based educational sessions, delivered every other week, in experimental school cafeterias during regularly scheduled lunch periods. Each session featured an eight-ounce smoothie prepared with frozen fruits selected for nutrient density, adolescent appeal, and consistency with NSLP crediting standards. See Table 1.

Each smoothie was nutrient-dense [57], providing 200–240 calories, 18 g of protein, two to four grams of fiber, and contributing several micronutrients (see Table 2). The nutrient composition of each smoothie recipe was estimated using Cronometer (Cronometer Inc., Revelstoke, BC, Canada), an accurate, comprehensive online nutrition analysis platform. Ingredients and portion sizes were entered based on the standardized recipes and matched to corresponding food items within the Cronometer database. Percent Daily Values (%DV) for vitamins and minerals were calculated via Cronometer using U.S. Daily Values based on a 2000-calorie reference diet, consistent with the 2020–2025 Dietary Guidelines for Americans.

The research team collaborated with school food service staff and prepared the smoothies on-site using standard school kitchen equipment, ensuring food safety and demonstrating the feasibility of future NSLP integration. Research assistants portioned smoothies into standardized eight-ounce servings and distributed them from a designated taste-test table in the cafeteria. To minimize disruption to the lunch routine, students were prompted to obtain their regular school lunch before approaching the smoothie table, and sessions typically began a few minutes after the start of lunch periods.

**Figure 1:**
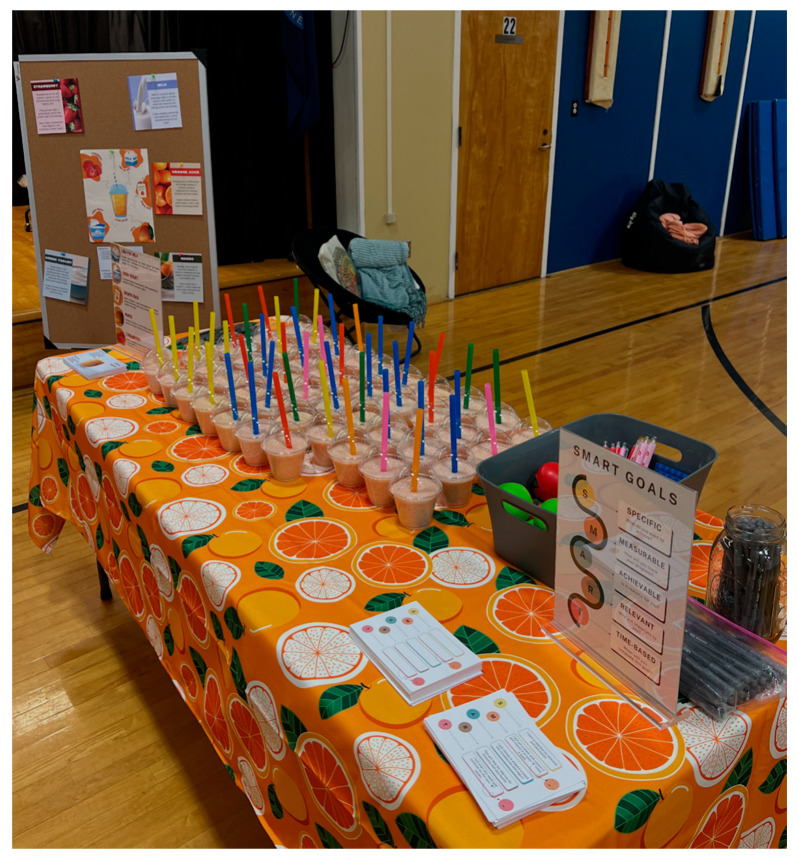
A HEALTHY (Helping Early Adolescents Live Their Healthiest Youth) smoothie tasting and nutrition education station incorporating Social Cognitive Theory, SMART (Specific, Measurable, Achievable, Relevant, Time Bound) goal setting, and Choice Architecture strategies to enhance visibility and appeal.

The smoothie tasting and nutrition education sessions were intentionally designed using SCT and Choice Architecture principles (Figure 1) [37,38,39,40,41,42,43]. These frameworks guided both the development of the sessions and the structure of the cafeteria-based experience. Social Cognitive Theory shaped the educational content by incorporating experiential tasting, peer engagement, self-efficacy building, and short skill-based activities such as SMART (Specific, Measurable, Achievable, Relevant, Time-Bound) goal setting [58]. Choice Architecture informed environmental design decisions such as placing the smoothie table in a highly visible location, serving smoothies in clear cups with colorful straws, displaying colorful educational infographics, and making the tasting process quick and convenient. Together, these elements were selected and implemented by the research team to create an environment that encouraged fruit selection, reinforced positive norms around fruit intake, and aligned with cafeteria operations.

Three trained RAs facilitated each smoothie tasting and nutrition education session. These assistants included Registered Dietitian Nutritionists (RDNs) and senior dietetic students with experience in community-based nutrition education. One RA delivered the brief verbal and visual education session, another distributed the smoothie and the corresponding recipe card, and the third guided students through SMART goal setting.

Students completed SMART goal cards aligned with that week’s educational theme or personal health goals and received small incentives (e.g., fruit-themed stress balls, pens, and stickers) to reinforce engagement and follow-through. At subsequent sessions, RAs prompted students to reflect on prior goals, reinforcing continuity and accountability. All recipes and education materials were reviewed for nutritional accuracy and appropriateness by three RDNs.

### 2.4. Instruments, Measures, and Procedures

At each school, five trained RAs collected data during the regularly scheduled lunch period on two consecutive days, both pre- and post-program. One comparison school was an exception due to scheduling constraints. Before fieldwork, RAs completed a standardized training led by the lead researcher that covered the study protocol for participant flow, digital tray photography, and skin carotenoid assessment procedures.

Upon entering the cafeteria, students whose parents had provided prior electronic consent checked in at an RA table, where we verified participation against a consent roster. We issued each consented student a paper identification (ID) card displaying their confidential ID number to carry throughout the lunch period. Students proceeded through the lunch line as usual.

Digital tray photography, a validated method that demonstrates strong agreement with weighted plate waste, was used to capture both selection and waste [59,60]. Immediately after food selection, an RA photographed each student’s tray to document the pre-consumption contents. At the end of lunch, RAs photographed each student’s tray again to capture post-consumption contents. During post-program assessments, smoothies were offered within the cafeteria salad bar, with photographs used to also capture smoothie consumption. By offering smoothies as part of the regular salad bar, smoothie selection, intake, and waste was able to be assessed when the program was no longer the potential driver.

The Veggie Meter^®^ (Longevity Link Crop., Salt Lake City, UT, USA), a validated, non-invasive, portable device that uses pressure-mediated wavelength reflection spectroscopy to measure epidermal carotenoids, was used to objectively characterize fruit consumption [50,51,52,53,54,55,61,62]. Approximately 10 min into the lunch period, an RA gathered consenting students in small groups, roughly two to five students at a time, for skin carotenoid assessments. Students sanitized their hands, and an RA obtained a single reading on the non-dominant ring finger. A single reading, rather than the average of three, was chosen to accommodate lunch-period time constraints. The Veggie Meter^®^ yields a score on a laptop interface from zero to 800, with higher scores indicating a greater carotenoid status, consistent with diets rich in diverse, colorful fruits and vegetables. Values were recorded in Microsoft Excel (Microsoft Corporation, Redmond, WA, USA).

Students also completed a brief paper-based survey informed by a previously published study evaluating students’ perceptions of the school food environment [63]. In the referenced study [63], the authors adapted the instrument to assess middle school-aged students’ perceptions of the school food environment across multiple domains, including food quality and appeal (e.g., “The food looks appealing”), interactions with food service staff (e.g., “The school lunchroom staff is friendly”), and the lunchroom atmosphere (e.g., “The school lunchroom is fun to hang out in”). The instrument also included items assessing students’ perceived weekly intake frequency of fruits, vegetables, and milk, which the authors noted were not previously validated [63].

For the present study, we used the perception and perceived intake items from the published instrument and added demographic items (age, grade, sex). The survey was administered in paper format by an RA at students’ lunch tables on one day of data collection, at pre- and post-program time points. All items were scored using a 5-point Likert scale ranging from “strongly disagree” (1) to “strongly agree” (5).

### 2.5. Data Analysis

Descriptive statistics were generated for all variables where appropriate. Due to student absences, logistical challenges, and differences in tray selections, sample sizes varied across outcomes.

Digital tray photographs were uploaded to a secure folder, matched pre- and post-meal for each student using their ID code, and subsequently organized by school. Tray items were coded according to the NSLP components (meat/meat alternatives, grains, fruits, vegetables, fluid milk). Because “combination foods” (e.g., a menu item that contains more than one meal component that cannot be separated) [64] could not always be disaggregated, meat/meat alternatives and grains were combined and coded as “entrées.”

Before analyzing digital tray photography, three RAs completed standardized Microsoft Excel-based (Microsoft Corporation, Redmond, WA, USA) training, created by the lead researcher, to ensure intra-rater reliability in visual waste estimation and familiarity with NSLP serving sizes. Each RA had to achieve at least 95% accuracy on a final quiz to pass the training.

Each RA independently scored each tray component using the validated six-point Comstock Indirect Visual Estimation Method (0 = none remaining to 5 = full portion remaining) [52]. Discrepancies were resolved through consensus review, and using Cohen’s inter-rater reliability guidelines, a kappa statistic was assigned for each tray item per student for all schools based on the RAs’ agreement (full RA agreement = 1.0, two-thirds RA agreement = 0.66, zero RA agreement = 0.0). As such, a 97% inter-rater reliability score was produced, indicating high agreement among RAs [65].

Day one and day two individual tray component scores were averaged to yield a single pre- and post-program mean for each component. All schools operated under the NSLP Offer Versus Serve (OVS) model, which allows students to choose from available meal components [66]. As a result, not all students selected all tray components equally. To account for this, dichotomous variables were created to indicate whether trays contained fruits, vegetables, fluid milk, or a smoothie.

Chi-square tests compared the proportions of students selecting fruit between groups at post-program, and McNemar’s tests assessed within-group changes from pre- to pos-program. A generalized linear mixed model (GLMM) accounted for school-level clustering, with fruit selection as a binary outcome, group (experimental versus comparison) as a fixed effect, and school as a random effect.

At the experimental schools, to examine whether students wasted less smoothie compared with the standard OVS fruit option, a paired *t*-test was conducted among trays that contained both items. Cohen’s *d* estimated effect sizes: small (*d* > 0.2), medium (*d* > 0.5), and large (*d* > 0.8) [67]. Estimated fruit consumption in cup equivalents was derived by reverse-coding waste scores (0 = 100% to 5 = 0%) and multiplying by 0.5 cup equivalents (the NSLP minimum fruit credit per serving).

Skin carotenoid levels were assessed for normality and averaged across days. Change scores (post–pre) were also calculated. Paired *t*-tests assessed within-group changes. Linear regression examined whether baseline levels predicted change. Finally, students were grouped into quartiles based on baseline skin carotenoid levels to evaluate whether those with the lowest initial scores showed greater improvement. This quartile-based approach aligns with methods used in prior Veggie Meter^®^ research in this population [68]. A repeated measures analysis of covariance (ANCOVA) compared post-program carotenoid levels between experimental and comparison students in the lowest quartile, controlling for baseline levels. Partial eta squared (η^2^) indicated effect size: small (η^2^ = 0.01), medium (η^2^ = 0.06), or large (η^2^ = 0.14) [67].

Survey data were analyzed descriptively. Any items containing negative wording were reverse-coded to ensure that higher scores indicated a positive response. A repeated-measures analysis of variance (ANOVA) evaluated between-group differences, accounting for time (pre vs. post).

All analyses were conducted in IBM SPSS Statistics for Mac (v29.02.0; IBM Corp., Armonk, NY, USA), with statistical significance set at *p* < 0.05.

## 3. Results

### 3.1. Participant Characteristics

A total of 149 students across five rural middle schools participated in the study (Table 3). Participants were evenly split by sex (49.2% male, 48.3% female, 2.3% chose not to answer), with an average age of 11.6 years (standard deviation = 1.5). Most students (72.1%, n = 93) were in grades sixth through eighth, with just over half from experimental schools (51.7%, n = 77) compared to comparison schools (48.3%, N = 72).

### 3.2. Fruit and Smoothie Selection

Chi-square revealed that at post-program, 73.0% of trays in experimental schools included fruit compared to 41.0% in comparison schools (χ^2^(1) = 9.95, *p* = 0.002). When smoothies were considered in addition to the standard fruit option, 98.3% of trays at experimental schools contained fruit compared with 41.0% of trays in comparison schools (χ^2^(1) = 41.66, *p* < 0.001). McNemar’s test showed that experimental schools significantly increased fruit selection from pre- to post-program (*p* = 0.004), whereas comparison schools demonstrated no significant change (*p* = 0.754). Table 4 highlights these findings.

The GLMM indicated that experimental trays had significantly higher odds of containing fruit at post-program than comparison trays, *F* (1,96) = 34.17, *p* < 0.001. Specifically, experimental students had 16.2 times higher odds of selecting fruit (OR = 16.22, 95% CI [6.30, 41.77], *p* < 0.001).

### 3.3. Fruit Waste and Estimated Consumption

Mean food waste scores (0 = no waste, 5 = full portion wasted) are presented in Table 5. At experimental schools, students who selected both fruit and a smoothie (n = 39) had significantly less smoothie waste than standard fruit waste, *t*(38) = −7.10, *p* < 0.001, *d* = 1.14. In contrast, mean fruit waste in comparison schools increased from pre- to post-program (0.9 ± 1.9 versus 1.5 ± 2.0). Across groups, smoothies were wasted far less than standard fruit items, and comparison school fruit waste was also higher than smoothie waste.

Based on reverse-coded waste scores and assuming a one-half-cup fruit serving, estimated consumption was ~0.15 cups for the standard fruit, compared with ~0.43 cups for smoothies.

### 3.4. Skin Carotenoid Levels

Skin carotenoid levels increased significantly from pre- to post-program in both groups. In experimental schools, lower baseline skin carotenoid levels significantly predicted greater improvement, *F* (1,62) = 32.183, *p* < 0.001, *R*^2^ = 0.34, B = −0.585. This relationship was not significant in comparison schools (*p* = 0.093). See Table 6.

Quartile analyses showed that experimental students in the lowest baseline quartile (Q1: <108) improved significantly more than comparison students, *F* (1,19) = 9.20, *p* = 0.007, partial η^2^ = 0.326. There were no significant differences in change scores for the other three quartiles (see Table 7).

### 3.5. Survey Findings

A repeated-measures ANOVA revealed only small, non-significant differences across groups on the survey items. Table 8 presents self-reported perceived intake of fruits, vegetables, and fluid milk from pre- to post-program among groups. These self-reported measures did not significantly change over time across groups.

## 4. Discussion

This study evaluated the HEALTHY program, which included four frozen-fruit smoothie-based educational sessions delivered every other week over eight weeks to promote fruit consumption and reduce fruit waste among rural adolescents. The primary aim of this study was to evaluate whether experimental schools demonstrated greater fruit intake, reduced fruit waste, and higher skin carotenoid levels than comparison schools. Preliminary findings provide support for this hypothesis, indicating higher fruit intake, reduced fruit waste, and improved skin carotenoid levels, particularly among students with the lowest baseline values, in experimental schools. Together, these results suggest that HEALTHY may be a promising theory-driven program for enhancing adolescent nutrition security, which requires not only access to nutritious foods but also consistent consumption, acceptability, and alignment with school food systems [12].

Previous school-based programs have reported mixed success in increasing fruit consumption, hindered by persistent waste despite greater fruit availability [69,70]. The present findings extend this literature, showing that frozen-fruit smoothies, when paired with behavioral frameworks, can shift both selection and consumption patterns. Although waste from the standard OVS fruit option increased post-program, this may represent a substitution effect in which students prioritized the smoothies over the traditional fruit served. Importantly, overall fruit consumption increased, indicating that frozen-fruit smoothies may support nutrition security by providing a more acceptable, nutrient-dense fruit source that adolescents are willing to consume.

Further, using frozen fruit ensured consistent quality and year-round availability [30,31], addressing barriers posed by rural geography and seasonality, while also aligning with adolescents’ preference for fresh-tasting options [22,23,24,25,26,27,71,72,73]. These findings are timely given that sugar-sweetened beverage consumption has risen by nearly one-third in the past decade [74]. Smoothies may represent a healthier alternative to sugar-sweetened beverages, though direct testing of this substitution is warranted.

To our knowledge, few studies have examined smoothies as a primary strategy for improving diet quality, and only one has been conducted within the school meal context. In the School Breakfast Program, one study found that adding smoothies substantially increased adolescents’ fruit intake [47]. Other smoothie-related studies have focused on clinical or adult populations, including patients receiving opioid agonist treatment, adults consuming smoothies as part of cardiometabolic risk reduction efforts, and studies characterizing the phytochemical profiles of fruit-based beverages [75,76,77,78]. These studies support the potential benefits of smoothies but do not fully address implementation within the NSLP, rural school settings, or outcomes such as food waste and skin carotenoid levels. By integrating frozen-fruit smoothies into routine school meals, pairing them with theory-driven provision and education, and evaluating both intake and objective biomarkers, HEALTHY extends this limited literature and provides novel evidence relevant to adolescent nutrition security.

The improvement in skin carotenoid levels, particularly among students who started the program with the lowest baseline scores, illustrates the potential for smoothie-based programming to advance nutrition security by reducing disparities in access to and consumption of nutrient-dense foods. By using quartile analyses rather than mean-only changes, this study applied an equity lens, demonstrating that adolescents with the poorest baseline scores experienced the most significant gains. Such findings mirror other nutrition-based programming, where the greatest benefits are experienced by those who are the most vulnerable [79,80], suggesting that smoothies may be a valuable strategy for addressing inequities in rural populations where fruit access is consistently limited.

Contextualizing the sample’s skin carotenoid levels highlights rural disparities in nutrition security. Baseline experimental values averaged 184.2 and increased to 196.2, while comparison school values rose from 165.7 to 193.8. These values fall within the lower range of pressure-mediated reflection spectroscopy measures in the literature for school-aged populations, which often span roughly 150 to 300 [51]. The comparatively lower values in the present study are therefore consistent with known disparities in fruit and vegetable intake among rural populations and underscore the relevance of using this biomarker in such communities.

It is noteworthy that carotenoid levels also improved in comparison schools. Seasonality may explain this pattern since pre-scores were collected in January, and post-scores were collected in May. Evidence from the literature has shown that skin carotenoid scores may decline during winter and increase during summer [52,81,82]. In the present study, pre-program assessments occurred in the coldest winter month, when carotenoid stores may be depleted [83,84], whereas post-program assessments coincided with early spring. This seasonal variation provides a potential explanation for the increases observed in both experimental and comparison groups. However, without dietary recall or broader biomarker tracking, this explanation remains speculative.

The dual application of Choice Architecture and SCT likely contributed to the observed positive outcomes. Choice Architecture strategies (e.g., clear cups, colorful straws, appealing table presentation) likely enhanced extrinsic motivation. At the same time, SCT-informed elements (e.g., goal setting, reinforcement, and peer modeling) supported intrinsic motivation and self-efficacy. Unlike many prior studies that relied solely on environmental nudges or education alone [33,34,35,36,38,39,40,41,42,43,44,46,47,48,49], HEALTHY integrated both, offering a novel theory-driven model. Prior research suggests that multilevel strategies are more effective than single-component strategies, and here [85], Choice Architecture and SCT likely amplified the effectiveness of one another.

Smoothie implementation also intersects with school meal policy and crediting standards. Under current United States Department of Agriculture (USDA) guidance, whole frozen fruit in smoothies must first be pureed and is then credited as juice, which may create a barrier to adoption despite its nutritional equivalence to fresh fruit [13,15,86]. Findings from this study underscore the importance of revisiting these standards so that evidence-based, waste-reducing, and student-preferred strategies can better support nutrition security within the NSLP. Importantly, implementation proved feasible within existing workflows when simple equipment, standardized recipes, and frozen fruit were used.

Beyond nutritional outcomes, the observed reduction in waste carries broader sustainability and cost implications. Food discarded in the NSLP represents a financial loss for already resource-limited communities and adds environmental burdens across the food system. Increasing consumption while reducing waste positions smoothies as a strategy that supports national goals for sustainable, health-promoting environments highlighted by Healthy People 2030 and by federal agencies, including the USDA, Centers for Disease Control and Prevention, and National Institutes of Health [87,88,89,90]. Therefore, presenting smoothies as a “win-win” that supports healthier diets, reduces costs, and limits waste may motivate nutrition directors responsible for balancing budgets, meeting federal meal standards, and satisfying students.

### Strengths and Limitations

Interpretation of these findings should consider several limitations. The quasi-experimental design without randomization limits causal inference, and the small number of participating schools and students reduces generalizability. The eight-week, biweekly design does not test long-term sustainability, and smoothies may have benefited from a novelty effect. As all five schools used the OVS model, not all students selected fruits or vegetables, which limited the number of observations available for some waste comparisons. Further, the smoothies sampled in this study were prepared with commercially purchased ingredients from Walmart rather than ingredients procured through USDA Foods in Schools or a similar food purchasing vendor. This may limit replicability for school nutrition programs unless standardized guidance is provided. Registered Dietitian Nutritionists and senior dietetic students leading the program may have influenced decisions, given evidence that peer-led programming often drives outcomes [91,92,93]. Additionally, individual-level systematic differences (e.g., socioeconomic characteristics or parental education) were not collected. Therefore, systematic differences cannot be assessed, and self-selection bias cannot be ruled out.

As such, future research should test HEALTHY in longer durations, across diverse schools, and with alternative facilitators. Comparative studies evaluating smoothies against other fruit-promotion strategies (e.g., sliced fruit, taste-tests, incentives) and individual- and district-level systematic and demographic differences would further clarify relative effectiveness.

Despite these limitations, the use of multiple objective measures, including digital tray photography and skin carotenoid levels strengthens confidence in the findings. Few school-based studies combine both intake and biomarker measures, making this a methodological strength. Importantly, the program was developed as student-centered [28,29], ensuring that the approach was feasible, relevant, and grounded in the reality of rural school food environments [94].

## 5. Conclusions

This study evaluated HEALTHY, a frozen-fruit smoothie-based nutrition education program implemented in rural middle schools to improve nutrition security using SCT and Choice Architecture. Following the programming, experimental schools demonstrated higher fruit selection and consumption relative to comparison schools. Although waste from the standard OVS fruit offering increased post-program, overall fruit consumption also increased, suggesting that students preferred the smoothie-based fruit offerings.

Skin carotenoid levels increased from pre- to post-program in both experimental and comparison schools. However, quartile analyses revealed that students in experimental schools with the lowest baseline carotenoid levels experienced the most significant increase, suggesting that the program may be especially effective for nutritionally vulnerable adolescents.

Together, these preliminary findings indicate that integrating frozen-fruit smoothies into the NSLP, when paired with behavior theory-based strategies and brief nutrition education, can increase fruit consumption, reduce fruit waste, and support improvements in objective dietary biomarkers, such as skin carotenoid levels, among nutritionally vulnerable rural adolescents. In this context, HEALTHY represents a policy-informing, equity-focused, and innovative strategy aligned with national priorities in nutrition security and sustainable food systems.

Future research is needed to assess sustainability, longer-term outcomes, seasonal influences, scalability across diverse school contexts, and the feasibility of adoption within current federal crediting standards to inform policy and practice effectively.

## Figures and Tables

**Table 1:** Weekly smoothie recipes and nutrition education themes in the HEALTHY (Helping Early Adolescents Live Their Healthiest Youth) school-based program.

Session	Smoothie Title	Educational Focus/Theme	Recipe
1	Power Play Shake	Fueling for sports performance, muscle recovery, and sustainable energy.	½ cup low-fat milk, ½ banana frozen, ½ cup mixed frozen berries, ½ cup Greek yogurt
2	Citrus Splash	Immunity and skin health.	½ cup low-fat milk, ¼ cup orange juice, ¼ cup frozen strawberries, ¼ cup frozen mango, ½ cup Greek yogurt
3	Hydration Hero	Natural hydration, reducing sugary beverage intake.	½ cup low-fat milk, ¼ cup orange juice, ¼ cup frozen peach, ¼ cup frozen cherry, ½ cup Greek yogurt
4	Maine Maple Blueberry	Benefits of local and seasonal eating.	½ cup low-fat milk, ½ cup frozen blueberries, ¼ cup pumpkin puree, ½ cup Greek yogurt, 1 tbsp maple syrup *

Notes: Smoothie recipes are based on an eight-ounce serving. For all recipes, one serving met at minimum the following National School Lunch Program reimbursable meal pattern components: 0.5 fluid milk, 1 fruit, 1 meat/meat alternative. * Considered an optional ingredient.

**Table 2:** Nutrient composition of the HEALTHY (Helping Early Adolescents Live Their Healthiest Youth) program smoothies (eight-ounce servings).

Nutrient	Power Play Shake	Citrus Splash	Hydration Hero	Maine Maple Blueberry
Calories (kcal)	240	210	210	200
Protein (g)	18	18	18	18
Total Fat (g)	4	4	4	4
Saturated Fat (g)	0	0	0	0
Total Carbohydrate (g)	30	26	24	21
Dietary Fiber (g)	4	2	4	4
Total Sugars (g)	0	0	0	0
Vitamin D (% DV)	10	10	10	10
Calcium (% DV)	30	40	40	35
Potassium (% DV)	25	25	25	25
Vitamin A (% DV)	15	15	15	80
Vitamin C (% DV)	25	70	30	4
Vitamin B12 (% DV)	25	25	25	25
Zinc (% DV)	10	8	8	15

Notes: Values reflect nutrients per eight-ounce serving as estimated using Cronometer (Cronometer Inc., Revelstoke, BC, Canada). Percent Daily Values (% DV) are based on a 2000-calorie diet.

**Table 3:** Demographic characteristics of students enrolled in the HEALTHY (Helping Early Adolescents Live Their Healthiest Youth) school-based program (N = 149).

Variable	% (n) or Mean (Standard Deviation)
Sex	
Male	49.2% (63)
Female	48.3% (62)
Chose not to answer	2.3% (3)
Age (Years)	11.63 (1.52), n = 128
Grade Level	
3rd	4.7% (6)
4th	15.5% (20)
5th	7.8% (10)
6th	20.9% (27)
7th	34.9% (45)
8th	16.3% (21)
School Designation	
Experimental	51.7% (77)
Comparison	48.3% (72)

Notes: Because of student absences and logistical challenges, sample sizes vary across outcomes.

**Table 4:** Proportion of trays containing standard fruit, smoothies, or other high-carotenoid items at pre- and post-program by school group.

	Vegetable	Standard Fruit	Smoothie	Standard Fruit and/or Smoothie	Fluid Milk
Experimental					
Pre	89.1%, n = 49	78.2%, n = 43	-	78.2%, n = 43	71.0%, n = 39
Post	66.1%, n = 39	73.0%, n = 43	91.5%, n = 54	98.3%, n = 58	35.6%, n = 31
Comparison					
Pre	71.9%, n = 23	37.5%, n = 12	-	-	62.5%, n = 20
Post	79.5%, n = 31	41.0%, n = 16	-	-	68.4%, n = 26

Notes: Values are presented as frequency, n. Because of student absences, logistical challenges, and variations in tray component selection, sample sizes vary across outcomes.

**Table 5:** Mean food waste scores for all tray components at pre- and post-program by school group.

	Entrée	Side	Vegetable	Standard Fruit	Smoothie	Total Fruit	Fluid Milk
Experimental							
Pre	1.1 (1.3)	1.4 (1.4)	2.3 (2.0)	2.0 (1.9)	-	-	0.3 (1.0)
Post	2.1 (1.5)	2.8 (2.0)	1.9 (1.9)	3.7 (1.9)	0.7 (1.2)	2.9 (2.5)	0.6 (1.6)
Comparison							
Pre	0.7 (1.3)	1.2 (1.50)	2.0 (1.8)	0.9 (1.9)	-	-	0.7 (1.8)
Post	1.0 (1.6)	2.4 (2.03)	2.4 (1.9)	1.5 (2.0)	-	-	0.4 (1.1)

Notes: Values are presented as mean (standard deviation). Because of student absences, logistical challenges, and variations in tray component selection, sample sizes vary across outcomes. Total Fruit: Average of standard fruit waste and smoothie waste. Scoring: 0 = None remained, 1 = ¼ portion remained, 2 = ½ portion remained, 3 = ¾ portion remained, 4 = Nearly full portion remained, 5 = Full portion remained.

**Table 6:** Overall skin carotenoid levels by school group.

	Mean (Standard Deviation), n
Experimental	
Pre	184.3 (85.9), n = 72
Post	196.2 (69.4), n = 68
Change	17.6 (66.1), n = 64
Comparison	
Pre	165.7 (77.6), n = 47
Post	193.8 (81.2), n = 53
Change	31.2 (65.5), n = 37

Notes: Because of student absences and logistical challenges, sample sizes vary across outcomes. Change scores only include participants with both baseline and post measurements.

**Table 7:** Changes in skin carotenoid levels by baseline quartiles by school group.

	Q1 (<108)	Q2 (108–<175)	Q3 (175–<230)	Q4 (>230)
Experimental				
Pre	79.6 (20.8), n = 17	145.8 (14.6), n = 19	205.0 (16.5, n = 14	285.3 (55.5), n = 22
Post	160.9 (55.6), n = 14	175.1 (68.8), n = 18	185.9 (37.4), n = 12	258.0 (54.0), n = 20
Change	85.7 (51.3), n = 14	28.7 (63.1), n = 18	−17.5 (47.7), n = 12	−19.0 (45.6), n = 20
Comparison				
Pre	76.3 (29.6), n = 13	133.6 (20.7), n = 10	203.3 (17.4), n = 17	286.2 (54.7), n = 7
Post	160.8 (32.3), n = 8	178.5 (65.0), n = 9	191.7 (61.0), n = 14	322.9 (109.2), n = 6
Change	78.0 (32.4), n = 8	47.8 (52.9), n = 9	−8.2 (71.8), n = 14	36.00 (55.9), n = 6

Notes: Values are presented as mean (standard deviation), n. Because of student absences and logistical challenges, sample sizes vary across outcomes. Change scores only include participants with both baseline and post measurements.

**Table 8:** Self-report fruit, milk, vegetable consumption at pre- and post-program by school group (N = 149).

	Fruit	Vegetable	Fluid Milk
Experimental			
Pre	1.9 (0.9), n = 57	2.1 (1.1), n = 57	3.1 (1.5), n = 57
Post	2.4 (1.0), n = 46	2.5 (0.9), n = 46	3.3 (1.5), n = 47
Comparison			
Pre	2.8 (1.5), n = 32	3.1 (1.1), n = 33	4.4 (0.9), n = 33
Post	2.7 (1.2), n = 50	3.3 (1.1), n = 50	4.2 (1.2), n = 50

Notes: Values are presented as mean (standard deviation), n. Because of student absences and logistical challenges, sample sizes vary. All items presented used a 5-point Likert scale, ranging from “strongly disagree” (1) to “strongly agree” (5).

## Data Availability

Data are available upon reasonable request made to the authors. The data are not publicly available due to privacy and ethical reasons.
